# Multiple sclerosis patients exhibit oral dysbiosis with decreased early colonizers and lower hypotaurine level

**DOI:** 10.1038/s41522-025-00787-7

**Published:** 2025-10-20

**Authors:** Rachel L. Fitzjerrells, Leeann Aguilar Meza, Meeta Yadav, Heena Olalde, Jemmie Hoang, Mishelle Paullus, Catherine Cherwin, Tracey A. Cho, Grant Brown, Sukirth M. Ganesan, Ashutosh K. Mangalam

**Affiliations:** 1 Interdisciplinary Graduate Program in Informatics, University of Iowa, Iowa City, IA USA; 2 College of Dentistry, University of Iowa, Iowa City, IA USA; 3 Holden Comprehensive Cancer Center, University of Iowa, Iowa City, IA USA; 4 Department of Pathology, Carver College of Medicine, University of Iowa, Iowa City, IA USA; 5 Department of Neurology, University of Iowa Health Care, Iowa City, IA USA; 6 College of Nursing University of Iowa, Iowa City, IA USA; 7 Department of Neurology, University of Iowa Health Care, Iowa City, IA USA; 8 Department of Biostatistics, University of Iowa, Iowa City, IA USA; 9 Department of Periodontics, University of Iowa College of Dentistry and Dental Clinics, Iowa City, IA USA; 10 University of Iowa, Iowa City, IA USA; 11 Iowa City VA Health Care System, Iowa City, IA USA

**Keywords:** Next-generation sequencing, Microbial communities, Metagenomics, Microbiome

## Abstract

Although gut microbiome dysbiosis is implicated in the pathobiology of multiple sclerosis (MS), the role of the oral microbiome (OM), the second largest microbiome, remains poorly understood. Additionally, while the salivary metabolome has been linked to other neurodegenerative diseases; its role in people with Relapsing-Remitting MS (pwRRMS), the most prevalent form of MS, is unknown. Combining shotgun metagenomics with untargeted metabolomics, we identified a reduced abundance of several early colonizing species including *Streptococcus* and *Actinomyces* in pwRRMS and an enrichment of bacteria with pathogenic potential including *Fusobacterium nucleatum, Porphyromonas gingivalis*, and several *Prevotella* species. pwRRMS had an altered metabolite profile including a decreased hypotaurine compared to healthy controls. Thus we report altered oral microbiome and metabolome in pwRRMS which might contribute to MS pathobiology. These findings offer potential microbiome-metabolome based diagnostic biomarkers for MS and pave the way for novel therapeutic interventions to improve disease management and patient outcomes.

## Introduction

Multiple sclerosis (MS) is a neuroinflammatory autoimmune disease that impacts roughly 2.9 million people worldwide^1^, with Relapsing-Remitting Multiple Sclerosis (RRMS), making up 85% of all MS cases^2^. The pathobiology of MS has been linked to both genetic and environmental factors including the HLA genes^3^, Vitamin D deficiency^4,5^, and the Epstein Barr Virus^6–9^. In recent years the gut microbiome has emerged as a potential environmental factor in RRMS^10^. We and others have shown that people with RRMS (pwRRMS) have dysbiotic gut microbiome^11–16^. Besides the gut, humans also harbor complex microbial communities at various other body sites, including the skin, oral cavity, respiratory tract, and urogenital tract.

The oral microbiome is the second most diverse microbiome, surpassed only by the gut, with over 700 bacterial species^17^. Oral dysbiosis is linked to local diseases such as dental caries and periodontitis but also neurodegenerative (i.e., Alzheimer’s^18–20^ and Acute Stroke^21^), and inflammatory diseases (i.e., atherosclerosis, pneumonia, rheumatoid arthritis, and heart disease^22^). To date, no study has jointly examined and correlated the oral microbiome and metabolome in RRMS patients. Although there are studies in oral microbiome profiling but these studies often have limitations such as low sample size or marker-based microbiome analysis (e.g., 16S rRNA gene sequencing) which may not capture the full microbial diversity or function. Additionally, there is a lack of data from North American populations.

Boullerne et al. found oral microbiome differences in twins discordant for MS (one with Clinically Isolated Syndrome, one with RRMS) and dysbiosis in both compared to prior published healthy control (HC) groups, but was limited by a sample size of two and lacked a matched control group^23^. A study by Troci et al., used 16S rRNA based microbiome analysis to show significant differences at the genus level^24^. Finally, Boussamet et al., also utilized 16S rRNA based microbiome analysis in 13 pwRRMS and 21 HCs to show oral dysbiosis in MS group^25^. They went a step further than previous studies by performing a flux balance analysis (FBA) that allowed them to predict potential microbiota-derived metabolites from the most prevalent taxa. However, since such predictions are less accurate than those derived from shotgun metagenomic sequencing, the results need to be validated through either shotgun metagenomic sequencing or direct metabolite measurement or both. Despite a good sample size (30 pwRRMS and 30 HCs), the study by Zangeneh et al., only profiled oral microbiota (using Denaturing gradient gel electrophoresis-DGGE) and not oral metabolite^26^. Recently, the connection between the salivary metabolome and other neurological diseases such as Parkinson’s and Alzheimer’s have been reported but its role in MS remains largely unknown.

Therefore to identify specific microbial species, bacterial metabolic pathways, and oral metabolites linked with the pathobiology of RRMS, we performed shotgun metagenomic sequencing and untargeted metabolomics on 50 pwRRMS and 50 HCs. We found many previously unidentified salivary alterations that reveal potential new targets with diagnostic and therapeutic potential. Additionally, our study underscores the importance of the oral microbiome and metabolome in the pathobiology of RRMS.

## Results

### The dysbiotic oral microbiome of pwRRMS

**Fig. 1:**
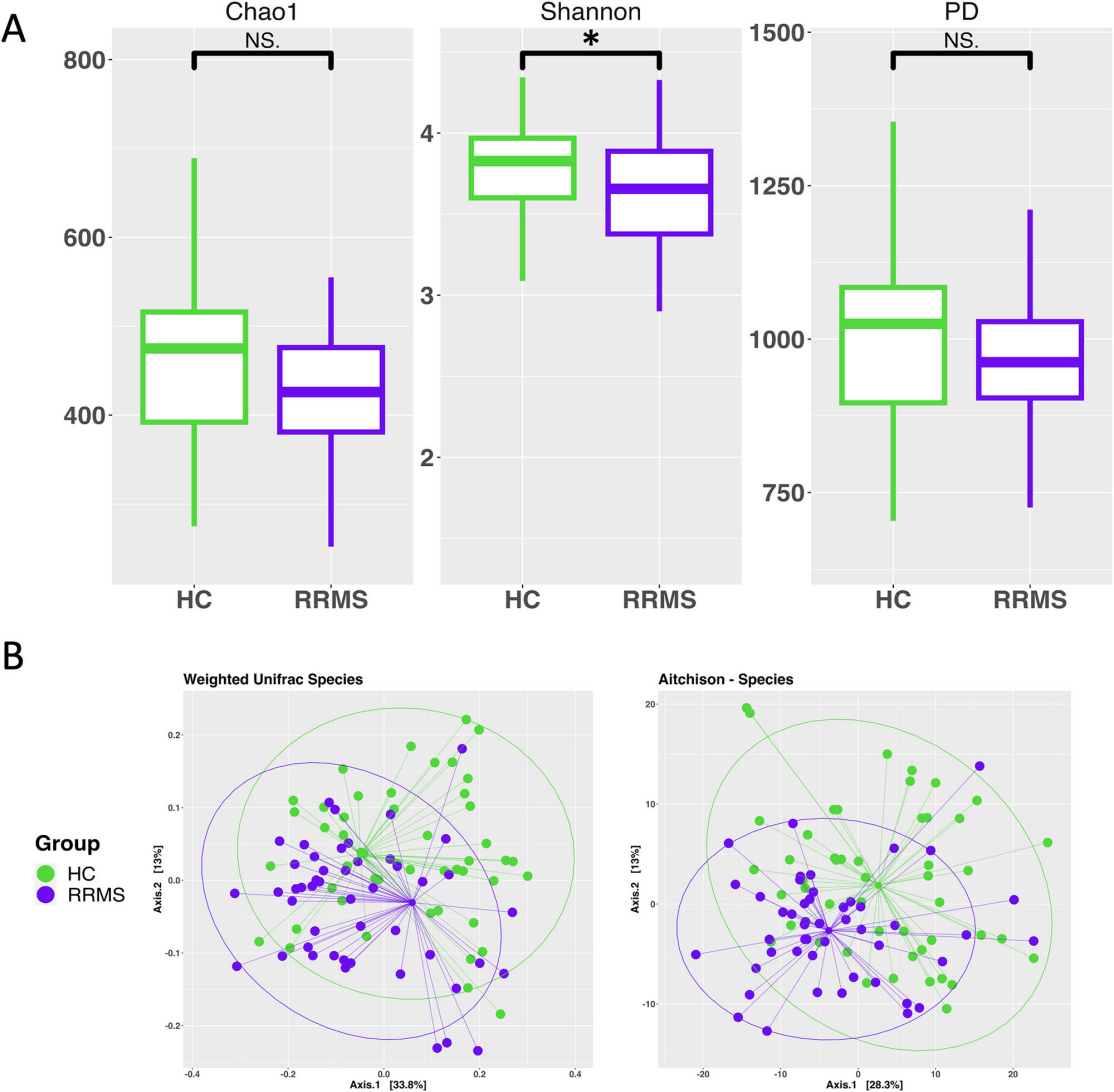
Alpha and beta diversity analyses of the oral microbiome in pwRRMS and HCs. **A** Three alpha diversity metrics utilized to assess individual diversity, Chao1, Shannon, and PD. Shannon diversity was significantly higher in HCs compared to pwRRMS. **B** The ecological assessment of beta diversity with the Weighted UniFrac metric (left) and the compositional approach of beta diversity with the Aitchison distance (right). Significance: N.S. >0.05, * <0.05, ** <0.01, *** <0.001.

To assess the oral microbiome profile of pwRRMS, we first considered potential confounding factors: sex, BMI, smoking history, and drug therapy. We did not identify significant differences to the oral microbiome based on these covariates in our cohort. See the “Cohort covariates” methods section for more detail. Shannon diversity (richness and evenness of microbes) analysis showed significantly higher alpha diversity in HCs compared to pwRRMS at the species level (Fig. 1A, *p* = 0.014). Chao1 (richness) and Faith’s Phylogenetic Diversity were not different between groups (*p* = 0.08, *p* = 0.1, respectively). Beta diversity with both ecological (Weighted UniFrac) and compositional (Aitchison) approaches showed distinct clustering of pwRRMS and HCs at the species level (Fig. 1B, *p* = 0.004, *p* = 1e−04, respectively). As for the viral and fungal components, neither were significant when assessing alpha diversity (Supplementary Fig. 1A, B), but for beta diversity they showed distinct cluster with the Aitchison metric (*p* = 0.2e−04, *p* = 0.005, respectively, Supplementary Fig. 1C, D). This highlights that for our cohort, the bacterial component of the oral microbiome is distinct in pwRRMS compared to HCs.

**Fig. 2:**
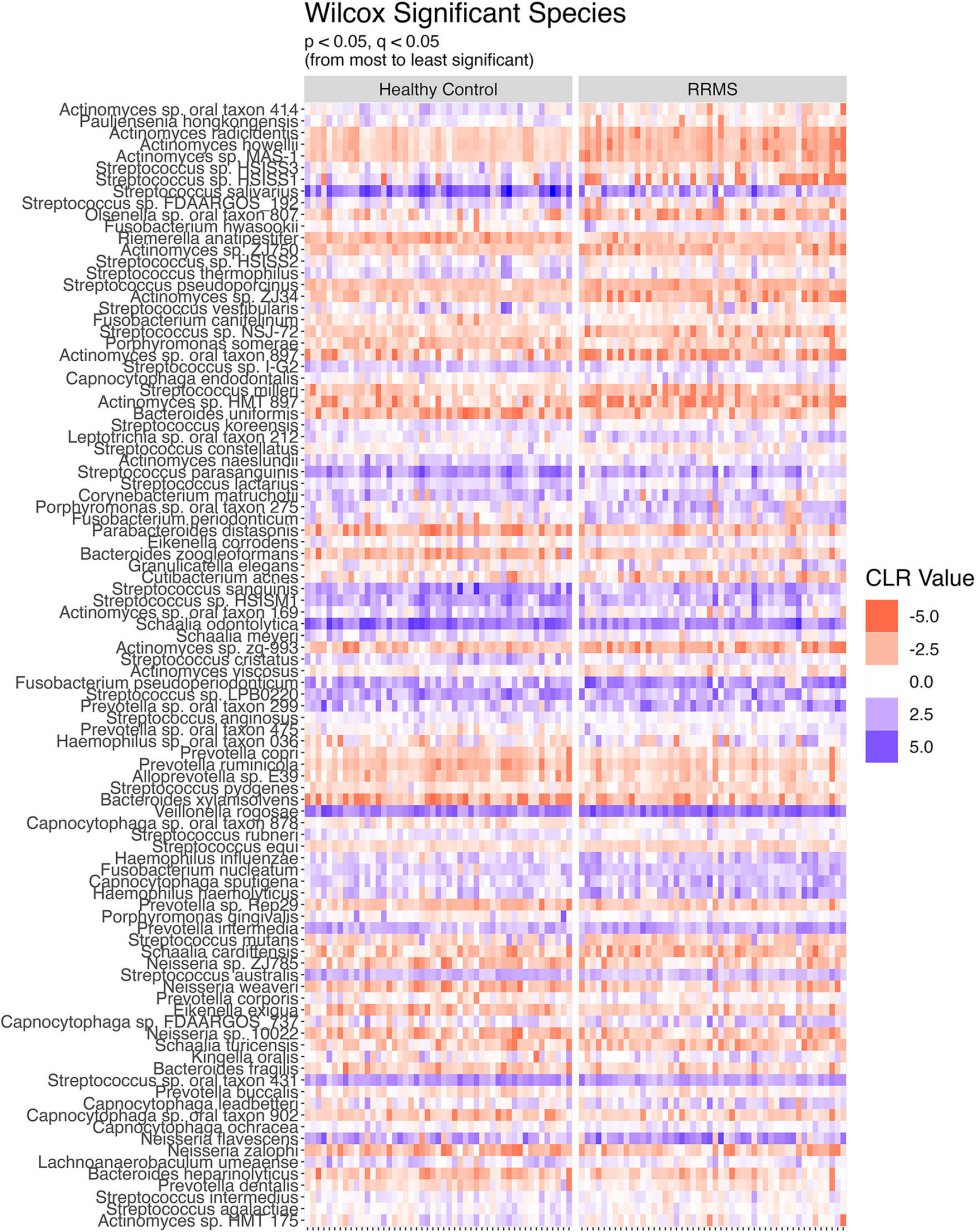
Differentially abundant bacterial species in pwRRMS compared to HCs. Each column represents an individual sample from each cohort. The coloring is based on the CLR value, a darker red value indicates a lower abundance of that species in the sample and a lower proportion of that sample’s total species load. A darker purple is the inverse: higher value and higher proportion. Species are ordered from most (top) to least (bottom) abundant.

At the individual microbe level 102 species were significantly altered in abundance in pwRRMS. Ninety-six bacteria (46 enriched, 50 lower in abundance) (Fig. 2), 5 viruses (2 enriched, 3 lower in abundance), and 1 fungal species (enriched) (Supplementary Table 1). Interestingly, all bacteria that were significantly reduced in RRMS were Gram positive whereas all but one significantly increased bacterium (*Granulicatella elegans*) were Gram negative in pwRRMS. Of the 96 altered bacterial species, the genera with the most species level changes were *Streptococcus* (28 species) and *Actinomyces* (13 species), all of which were lower in pwRRMS. Whereas *Prevotella* genus was increased in pwRRMS, specifically *P. dentalis, P. buccalis, P. intermedia*, and *P. copri*^27^, which have been linked to periodontitis. Of note, *Porphyromonas gingivalis*, a key pathogen implicated in periodontitis^27^, exhibited significantly higher abundance in pwRRMS. Additionally, *Fusobacterium nucleatum*, a bacterial species commonly associated with oral and systemic diseases^27,28^, was increased in pwRRMS.

**Fig. 3:**
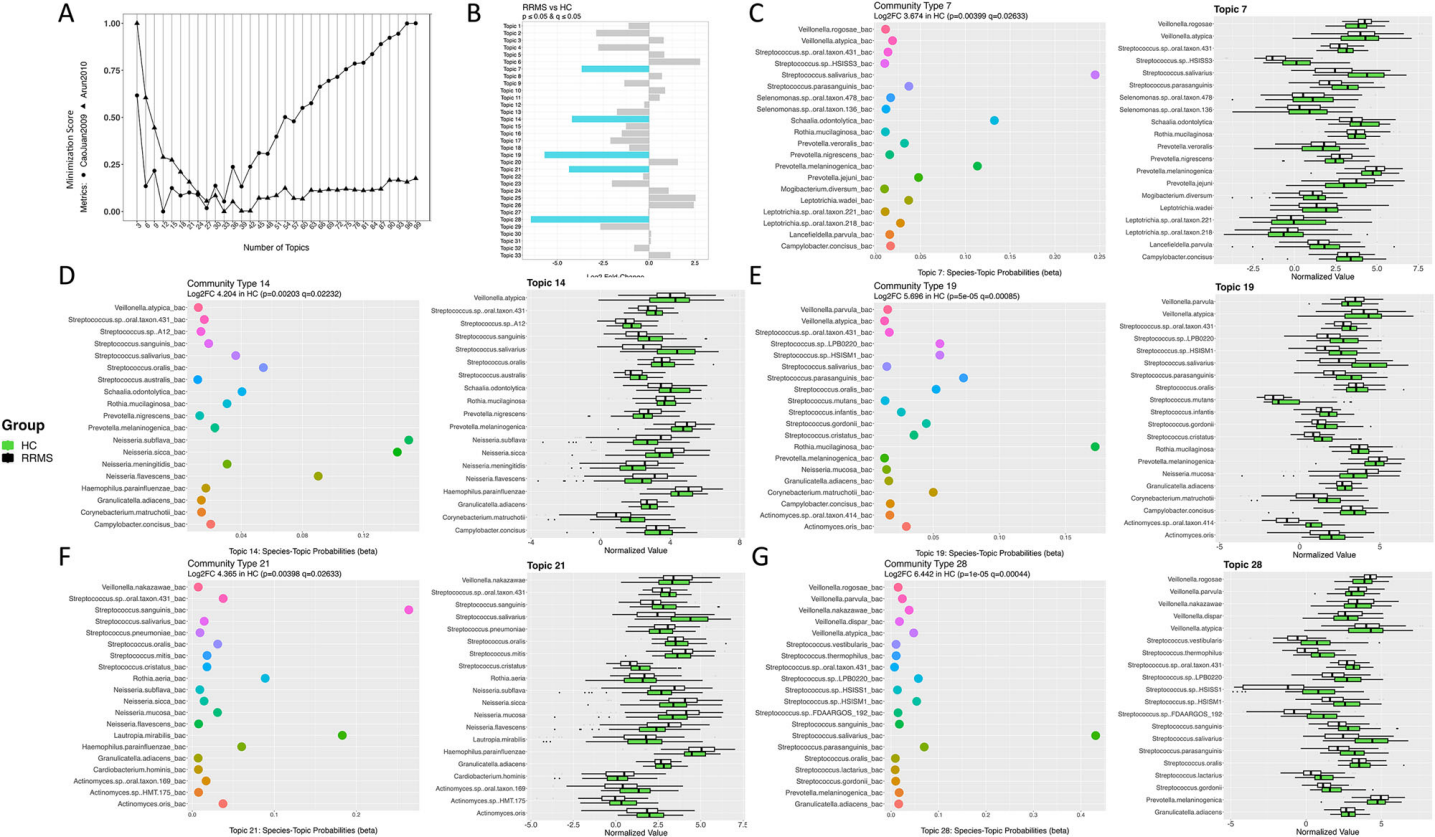
Topic modeling reveals distinct microbial signatures associated with pwRRMS and HCs. **A** The ideal topic number, 33, identified using minimization metrics as per Arun2010^62^ and CaoJuan2009^63^. The *y*-axis represents the minimization score calculated by each metric based on the topic number (*x*-axis). **B** 5 of the 33 topics were significantly more often assigned to HCs (in blue). **C**–**G** (left) top 20 bacteria most often assigned to significant topic with *x*-axis representing the probability of species assignment and (right) the corresponding normalized abundance of each of the assigned bacteria for reference.

To assess the community of microbes identified in pwRRMS or HCs, we performed topic modeling with all bacteria, viral, and fungal species. Topic modeling is an unsupervised machine learning approach that looks for patterns or themes within data^29^, here we analyzed for bacteria often found together (communities) in our samples. Among the 33 topics assessed, only bacteria were found to be highly assigned. Of these 33 topics (Fig. 3A), there were 5 microbial communities that were more often assigned to HCs than pwRRMS (Fig. 3B). Across many HC communities was the assignment of multiple species of *Streptococcus, Veillonella*, and/or *Actinomyces* (Topics 17, 14, 19, 21, and 28; Fig. 3C–G). The topic with the largest Log2 Fold-Change in assignment to HCs was Topic 28 and this topic was most often assigned *S. salivarius*, an alkali producing species^30^ (Fig. 3G). *P. melaninogenica*, which was higher in abundance in patients with Rheumatoid Arthritis and Alzheimer’s Disease^31,32^, was assigned to Topic 28, but its abundance was overall lower in HCs (n.s.). Additionally, two species known to aggregate and alter one another’s gene expression^33^, *V. parvula* and *S. gordonii*, were assigned to Topic 28. These two bacteria were also found together in HC Topic 19 (Fig. 1E).

Thus, we identified that pwRRMS have a distinct oral microbiome from that of HCs, an enrichment or reduced abundance of specific bacterial species, and, with topic modeling, we identified bacterial community-level patterns unique to healthy controls, suggesting a potential loss of beneficial microbial communities in pwRRMS.

### Functional properties of the oral bacteriome in pwRRMS

**Fig. 4:**
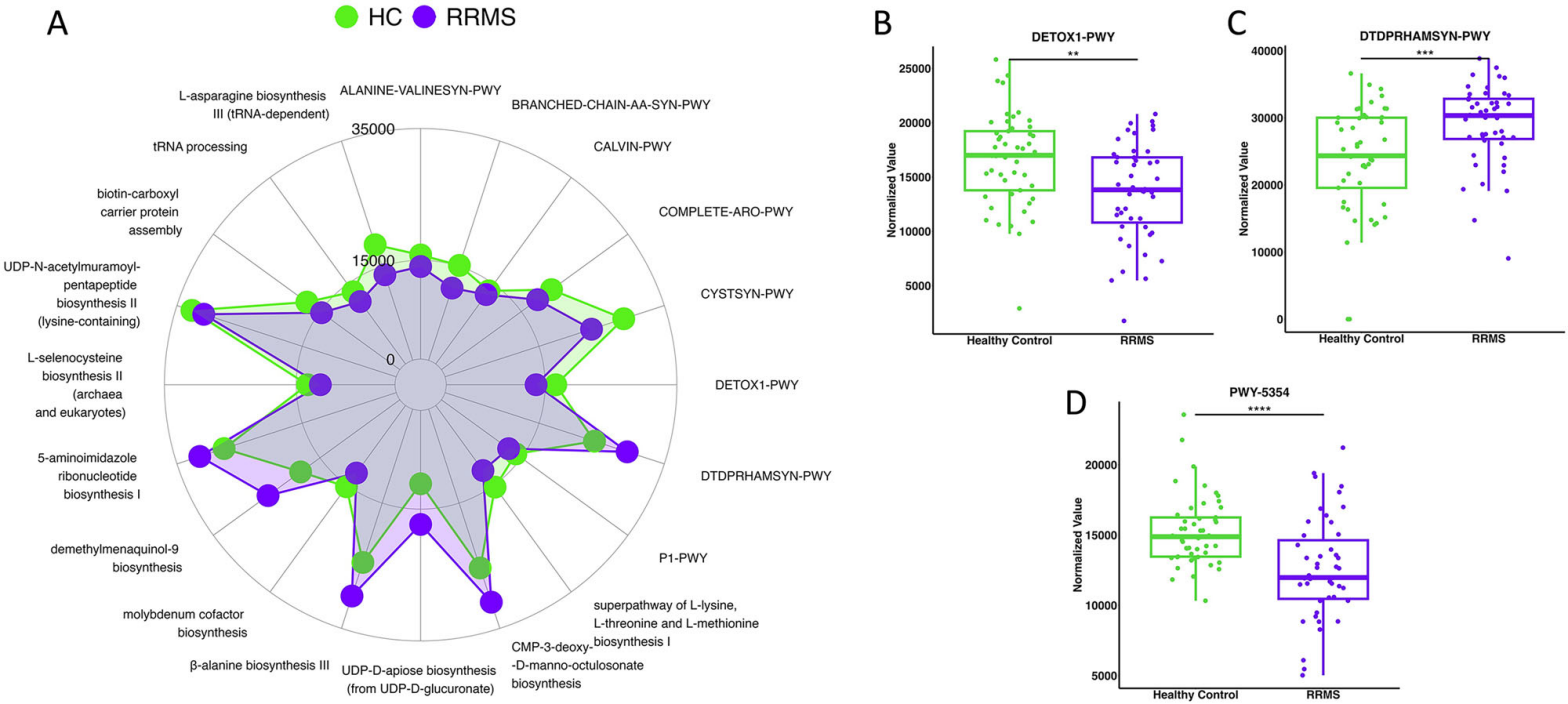
Functional pathway alterations in the oral microbiome of pwRRMS. **A** 20 functional pathways were significantly altered between RRMS and HCs. Points further from the center indicate a higher abundance. **B** Superoxide radicals degradation pathway. **C** dTDP-β-L-rhamnose Biosynthesis pathway. **D** Molybdenum cofactor synthesis pathway.

Next, we assessed the associated functional pathways contributed by the bacteria. There were 20 functional pathways altered in pwRRMS, 6 enriched and 14 reduced (Fig. 4A). “DETOX1-PWY” or Superoxide Radicals Degradation, which produces hydrogen peroxide and water^34^, preventing the growth of acidophiles^30^, was significantly lower in pwRRMS (Fig. 4B). A function increased in pwRRMS was “DTDPRHAMSYN-PWY” or dTDP-β-L-rhamnose Biosynthesis (Fig. 4C), a pathway utilized by both Gram positive and Gram negative bacteria pathogens to build their cell walls^35,36^. As multiple Gram-negative species such as *Prevotella, Veillinola, and Fuscobacetrium species* were increased in pwRRMS, our data points towards an important role of L-rhamanose in pathobiology of MS potentially through increased immunostimulatory LPS activity. Additionally, “PWY-5354” or Molybdenum Cofactor Synthesis, which is involved in converting taurine to hypotaurine, was significantly lower in pwRRMS (Fig. 4D). Thus, the functional capacity of the oral bacteria in pwRRMS is distinct from HCs, specifically the decrease of functional pathways (DETOX1-PWY, PWY-5354) linked with homeostasis.

### Oral metabolome alterations in pwRRMS

**Fig. 5:**
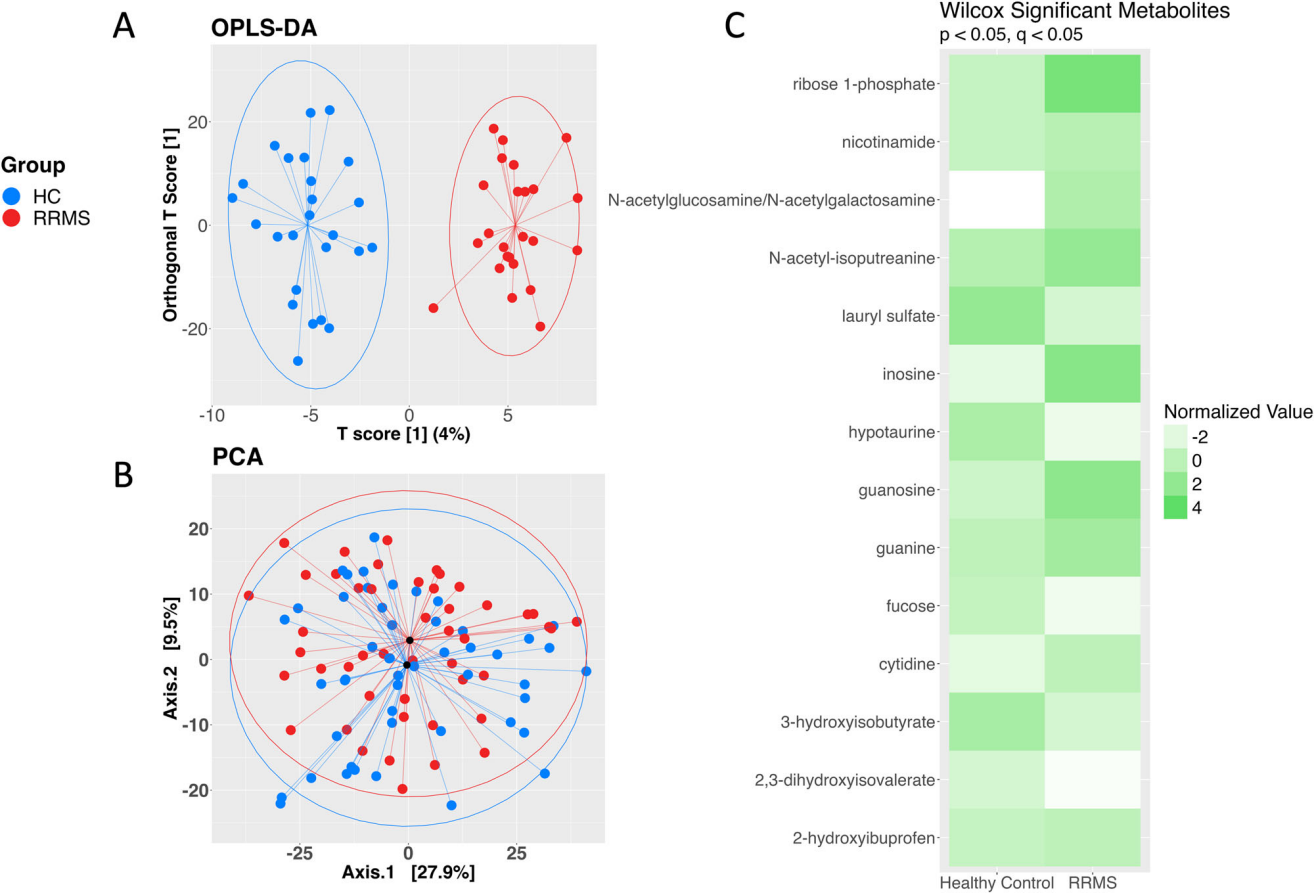
Oral metabolome alterations distinguish pwRRMS from HCs. **A** Orthogonal partial least square-discriminant analysis, a supervised approach, distinctly clusters the oral metabolome of pwRRMS from HCs. **B** Principal component analysis shows oral metabolome is not dissimilar between pwRRMS and HCs. **C** 14 metabolites were significantly altered in pwRRMS compared to HCs.

We found no significant association between the oral metabolome and potential confounding factors (sex, BMI, smoking history, drug therapy) in our pwRRMS cohort. See the “Cohort covariates” methods section for more detail. Utilizing Orthogonal Partial Least Squares- Discriminant Analysis (OPLS-DA) to compare oral metabolome between pwRRMS and HCs, we observed clear clustering between these two groups and the test data achieved ~75% prediction accuracy (Fig. 5A). Although our OPLS-DA model fit the training data well (*R*^2^ = 0.91), its low *Q*^2^ value (0.455) suggests potential overfitting and limited generalizability. Therefore, we also performed Principle Component Analysis (PCA), an unsupervised approach that focuses on explaining variance rather than separating groups. The samples did not distinctly cluster with this approach (Fig. 5B, *p* = 0.051). At the individual metabolite level, in pwRRMS, 5 metabolites were lower in abundance: hypotaurine, lauryl sulfate, fucose, 3-hydroxyisobutyrate, and 2,3-dihydroxyiosvalerate and 9 were increased in abundance: ribose 1-phosphate, nicotinamide, N-acetylglucosamine/N-acetylgalactosamine, N-acetyl-isoputreanine, inosine, guanosine, guanine, cytidine, and 2-hydroxiosbutyrate (Fig. 5C).

**Fig. 6:**
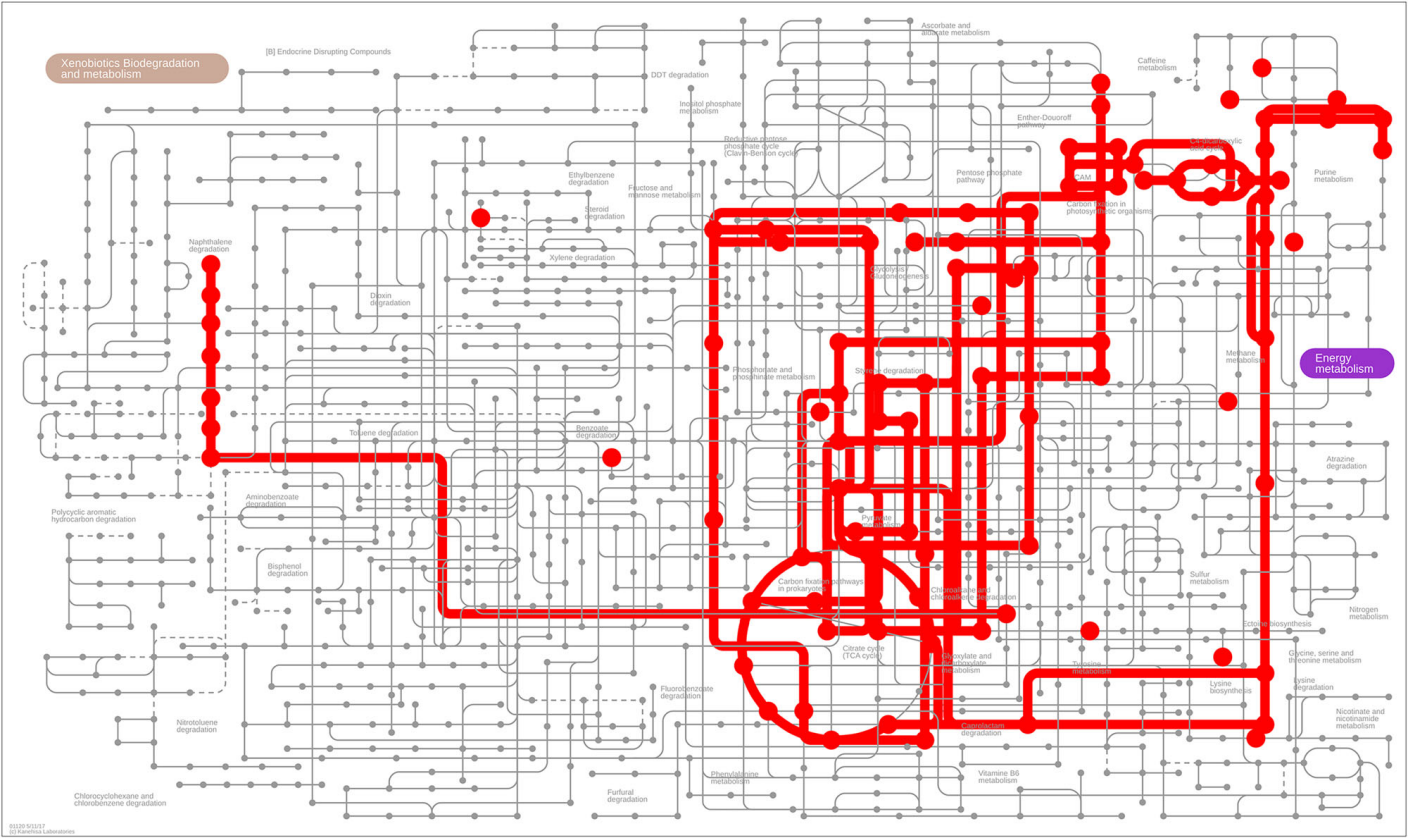
Microbiome-derived metabolites mapped to metabolic pathways. These metabolites were supplied to *pathwyas.embl.de* in order to find corresponding modules. 18 of these metabolites could be confirmed and their corresponding pathways are highlighted in red.

Using AMON analysis^37^, which differentiate mammalian-derived metabolites from microbial-derived metabolites, we identified that 55 metabolites from potential microbial origin and 33 metabolites as human origin (Supplementary Table 2). Utilizing *pathways.embl.de*, we identified a total of 228 module hits to our microbial metabolites (Fig. 6). Pathways with many hits included “Citrate cycle (TCA Cycle)”, “Glyoxylate and dicarboxylate metabolism”, “Chloroalkane and chloroalkene degradation”, “Pyruvate metabolism”, “Styrene degradation”, “Phosphorate and phosphinate metabolism”, “Glycolysis/Glucogenesis”, “Tyrosine biosynthesis”, and “Lysine biosynthesis”.

**Fig. 7:**
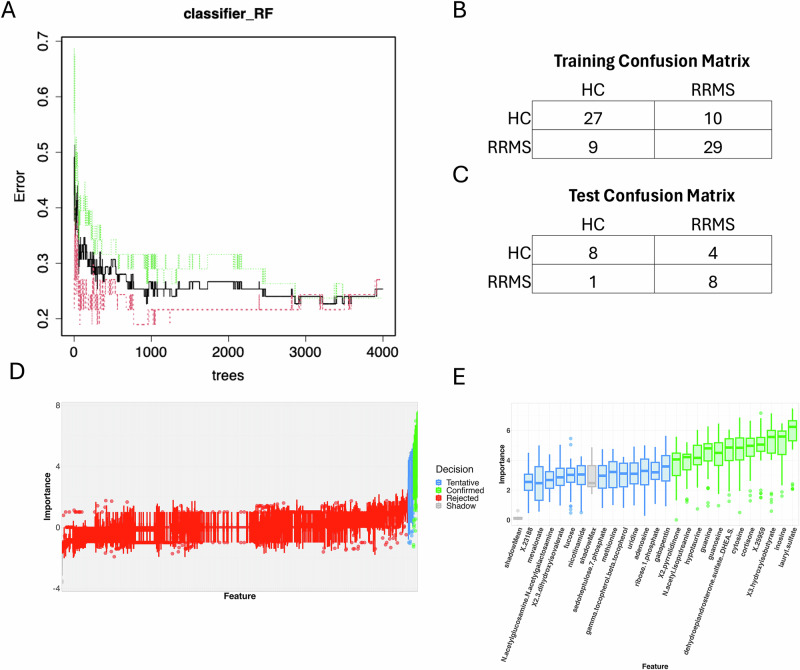
Random Forest–based machine learning identifies discriminatory metabolite features in pwRRMS. **A** Random forest classifier built with 4000 trees. **B** Training data confusion matrix showing an OOB error rate of 25.33%. **C** Testing data confusion matrix showing correct labeling of 67% HCs and 89% of pwRRMS. **D** Boruta importance decision of all metabolites. **E** All features not rejected by Boruta (importance >1.5).

Next, random forest analysis was employed to identify discriminatory metabolites based on their ability to differentiate pwRRMS from HCs. We performed this analysis with 4000 trees which resulted in an out-of-bag (OOB) error rate of 25.33% (Fig. 7A, B). Our model labeled test data correctly at about 67% accuracy in HCs and 89% accuracy in pwRRMS (Fig. 7C). Boruta identified 12 significant metabolites in distinguishing pwRRMS and HCs: hypotaurine, lauryl sulfate, inosine, hydroxyisobutyrate, X-25959, cortisone, cytosine, dehydroepiandrosterone sulfate (DHEA), guanosine, guanine, N-acetyl-isoputreanine, and pyrrolidinone (Fig. 7D, E). Overall, both Wilcoxon and random forest identified several significantly altered metabolites, and interestingly, many metabolites were identified as only produced by the microbiome. This indicates that the derived metabolites of the altered oral microbiome of pwRRMS likely play a role in the pathobiology of RRMS.

### Correlations of the oral microbiome and metabolome in pwRRMS

**Fig. 8:**
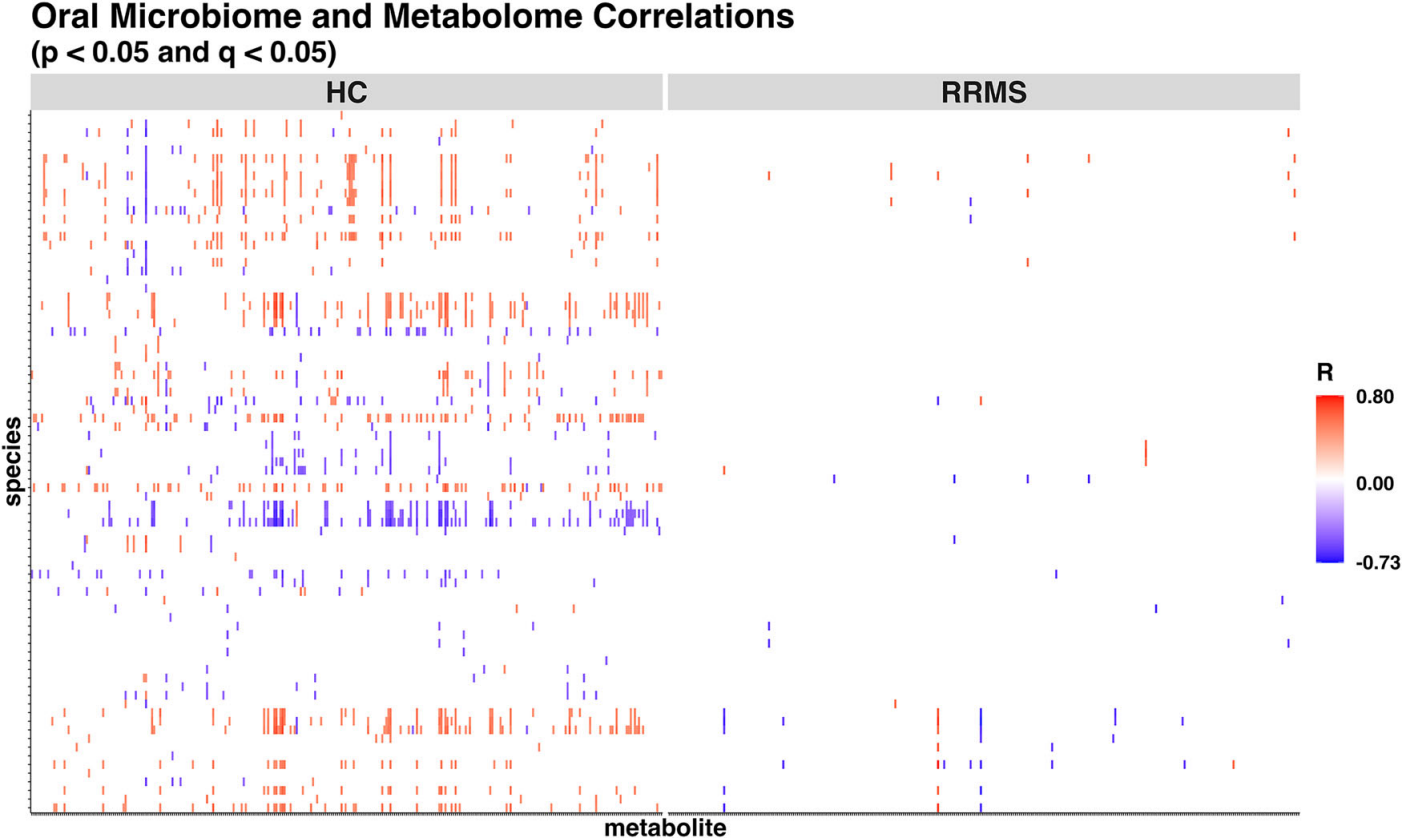
Significant spearman correlations between the oral metabolites (*x*-axis) and significant oral bacteria (*y*-axis). Positive correlations in red, negative correlations in blue.

In our spearman correlation analysis between the significant oral bacteria and the salivary metabolites, we identified 65 significant correlations in pwRRMS (28 positive, 37 negative) and 1339 significant correlations in HCs (891 positive, 448 negative) (Fig. 8 and Supplementary Data 1). In the HC cohort there were three significant correlations with Hypotaurine: *Streptococcus sp. HSISS1* (*R* = 0.467)*, Actinomyces sp. ZJ34* (*R* = 0.476), and *Granulicatella elegans* (*R* = −0.536) (Supplementary Fig. 2A). There were also two significant correlations with Taurine: *Kingella oralis* (*R* = 0.472) and *Actinomyces naeslundii* (*R* = 0.473) (Supplementary Fig. 2B). These results reveal more than 20 times as many significant correlations in HCs compared to pwRRMS, with significant hypotaurine and taurine correlations only identified in HCs. These findings indicate that the associations between oral bacteria and salivary metabolites differ between HC and pwRRMS groups, with HCs exhibiting many more significant relationships. This may reflect a more coordinated or integrated microbial–metabolite network in HCs, whereas such interactions appear to be disrupted or altered in pwRRMS.

## Discussion

While the oral microbiome and metabolome is implicated in the pathobiology of neurodegenerative diseases^23–26^, the role of the oral microbiome and metabolome in MS remains poorly understood. Our study addresses this gap by providing a comprehensive analysis of the oral microbiome and metabolome in pwRRMS, showing significant alterations in bacterial species and metabolite levels. These findings offer new insights into the complex interplay between the oral microbiome and host physiology in MS, with implications for disease mechanisms and potential therapeutic targets.

Among the altered bacteria in RRMS, the genera with the most species levels changes were *Streptococcus* and *Actinomyces* species, all of which were decreased in pwRRMS compared to HCs. These genera are early colonizers of the oral cavity and are important building blocks of the oral microbiome, as they produce numerous adhesive molecules that facilitate the colonization of various oral tissues^30,38^. Thus, reduced abundance of *Streptococcus* species in pwRRMS aligns with previous findings from other oral microbiome studies which showed an enrichment of three *Streptococcus* species- *S. parasanguinis, S. sanguinis*, and *S. intermedius*^30,39,40^ in HC. However, the lower abundance of the *Actinomyces* genus in pwRRMS contrasted with findings by Zangeneh et al., who reported higher levels of *Actinomyces* in individuals with MS compared to HC in their study of the oral microbiome^26^. The difference in *Actinomyces* abundance between studies may be attributed to factors such as bacterial profiling methods and geographical variation. Zangeneh et al. used DGGE, which detects selected bacterial genera, whereas our study used shotgun sequencing, a method capable of identifying hundreds of bacterial species. Additionally, the difference in geographical location (Iran vs. the United States) may affect microbiome composition due to variations in genetic backgrounds and environmental exposure*s*^41^. However, future studies involving a large sample size from different geographical regions are needed to better understand changes to *Actinomyces* species in the saliva microbiome of pwRRMS.

Both *Streptococcus* and *Actinomyces* are Gram positive bacteria, and all bacteria significantly increased in pwRRMS were Gram-negative (except for *G. elegans*). Gram-negative bacteria contain lipopolysaccharide (LPS) in their outer membrane, which can trigger a pro-inflammatory immune responses^42^. Many of these bacteria thrive in low-oxygen environments, such as periodontal pockets or inflamed tissues^43,44^, which may be relevant in the context of MS-related oral inflammation. Additionally, increased L-rhamnose in pwRRMS suggest potential pro-inflammatory role of this pathway in the pathobiology of MS. While *Streptococcus* is a known producer of L-rhamnose, this sugar is also found in the LPS of Gram-negative bacteria^35,36^. In our data, we observed an increase in taxa such as *Prevotella*, *Veillonella*, and *Fusobacterium*, all of which can utilize this pathway. Since humans do not synthesize L-rhamnose^35^, its elevated levels likely reflect microbial shifts toward these Gram-negative taxa, which may contribute to inflammation via LPS-mediated immune modulation. Overall, our findings suggest that L-rhamnose metabolism by non-streptococcal taxa may play a role in MS and warrants further investigation. Thus, the oral dysbiosis observed in pwRRMS is characterized by an overall increase in Gram-negative bacteria and a reduction in Gram-positive early colonizing bacteria.

Our metabolomic analysis revealed a significant reduction in salivary hypotaurine, an intermediate in the biosynthesis of taurine, in individuals with RRMS. Interestingly, the cysteine synthesis pathway (CYSTSYN-PWY), which provides the precursor for taurine biosynthesis, was also significantly downregulated in pwRRMS. Considering the critical role of taurine in neurological development and myelin sheath maintenance^45,46^, this downregulation of hypotaurine and the cysteine synthesis pathway could contribute to the pathobiology of MS. In fact, in in-vitro studies, the combination of remyelination therapies with endogenous taurine has resulted in increased remyelination^47^. Additionally, a mouse aging study showed serum taurine levels significantly decline with age. However, taurine supplementation improved survival and bone, brain, muscle, and immune function^48^. Human studies correlating metabolites with clinical risk factors linked poor health to lower taurine levels as well^48^. This aligns with the lower taurine, hypotaurine, and N-acetyltaurine levels in our RRMS cohort (only hypotaurine was significantly lower). Interestingly, *V. parvula* and *S. gordonii* were found in two of our significant HC community topics and their coaggregation can influence Taurine and Hypotaurine Metabolism^33^. Although these organisms were not significantly altered in abundance, they were a part of a healthy oral microbiome based on our topic modeling analysis. There were many more correlations between the microbiome and metabolome in HCs, exceeding that of pwRRMS by over 20 times. Considering hypotaurine correlations, *Granulicatella elegans*, a common oral microbe, was negatively correlated with hypotaurine in HCs. *G. elegans* had been linked to acute pulpitis, or inflammation of the dental pulp^49^. Taurine was also positively correlated with *Actinomyces naeslundii* in HCs. The increase in beneficial taurine in HCs along with the increase in *A. naeslundii*, suggest a potential symbiotic role for *A. naeslundii* as it can play a crucial role in the formation of a healthy early biofilm by adhering to other bacteria, epithelial surfaces, teeth, and collagen^38^. Our analyses consistently highlight a link between reduced hypotaurine levels and RRMS, suggesting that salivary hypotaurine may serve as both a novel therapeutic target and a potential biomarker for RRMS.

This study significantly advances our understanding of the oral microbiome and metabolome in RRMS. However, there are several limitations that should be addressed in future studies including the inclusion of fully matched cohorts, a more diverse demographic, longitudinal analysis, and the application of advanced bioinformatic techniques to better identify the origins of metabolites.

One key limitation of our study is the lack of clinical periodontal assessments, which prevented us from directly evaluating the impact of periodontitis on the oral microbiome and its potential association with RRMS. Based on previously reported periodontitis prevalence rates^50^, we estimate that a subset of our participants may have had periodontitis. However, our study was underpowered to detect any meaningful correlation between periodontitis and the microbiome. Future studies with larger sample sizes and direct periodontal assessments will be needed to clarify the relationship between periodontitis, the oral microbiome, and MS pathobiology.

In conclusion, our comprehensive analysis shows significant alterations in the oral microbiome of pwRRMS, specifically a depletion of key early colonizers *Streptococcus* and *Actinomyces*. Furthermore, untargeted metabolomics identified hypotaurine as a potential salivary biomarker for RRMS, consistently differentiating patients from HCs across multiple analysis approaches. Collectively, our findings underscore the critical interplay between the oral microbiome, metabolome, and disease pathogenesis in RRMS. These findings, while promising, should be interpreted with caution due to the lack of periodontal data and may reflect an indirect association rather than a direct link between the oral microbiome and MS.

## Methods

### Patient recruitment and demographics

This study was approved by the University of Iowa Institutional Review Board (Iowa City, IA, USA) and performed in accordance with the ethical standards as laid down in the 1964 Declaration of Helsinki and its later amendments or comparable ethical standards. Informed consent was obtained from all individual participants included in the study. Adult patients diagnosed with RRMS were recruited from the University of Iowa Neurology Clinic (*n* = 50). Some individuals were on disease modifying therapies (Table 1). Adults without MS or other autoimmune diseases were recruited as HCs (*n* = 50) through the University of Iowa. Inclusion/exclusion criteria for all individuals were: never smoked or quit smoking at least 10 years ago (the time it takes the oral microbiome to resemble that of someone who has never smoked^51^) and no antibiotics within the last 3 months.

**Table 1:** Cohort demographics

	HC (*n* = 49^a^)	RRMS (*n* = 48^a^)	Statistical notes
Age (mean ± SD)	43.08 ± 18.66	42.02 ± 8.64	t.test, *p* = 0.7197
BMI (mean ± SD)	26.21 ± 5.36	28.68 ± 5.16	Linear regression revealed BMI did not significantly impact the oral microbiome of our cohort
Sex	F = 28	F = 43	Linear regression revealed sex did not significantly impact the oral microbiome of our cohort
	M = 21	M = 5	
Race	White = 38	White = 46	
	Asian = 8	Multiracial/two or more races = 1	
	Black or African American = 1	Black or African American = 1	
	American Indian or Alaskan Native = 1		
	Prefer not to answer = 1		
Smoking status	Never smoked = 49	Never smoked = 43	Preliminary analysis revealed smoking history did not impact the oral microbiome of our MS cohort
	Quit 10+ years ago = 0	Quit 10+ years ago = 5	
Drug therapy		Ocrelizumab = 20	Preliminary analysis revealed drug therapy did not impact the oral microbiome of our MS cohort
		Glatiramer acetate = 9	
		Dimethyl fumarate = 6	
		None = 6	
		Interferon beta-1a (IM) = 3	
		Ofatumumab = 2	
		Interferon beta-1a (SC) = 1	
		Diroximel fumarate = 1	

Demographics of individuals used in analysis (post-filtering).

*SD* standard deviation.

^a^We recruited 50 pwRRMS and 50 HCs however, two RRMS samples were removed due to low DNA content and one HC was removed (see “Cohort covariates” methods section for more details).

### Saliva sample collection, DNA extraction, shotgun sequencing and untargeted metabolomics

Saliva samples were collected with the Thermo Scientific SpeciMAX Saliva Collection Kit during patient clinic visit or priority mailed. Samples were then immediately brought or delivered to our laboratory, stored at -80°C, and remained frozen until DNA extraction. We utilized Qiagen DNeasy PowerLyser PowerSoil Kit (Qiagen, Germantown, MD) for DNA extraction. We followed the manufacturer’s instructions by performing the bead-beating step (PowerLyzer 24 Homogenizer, Omni International, USA). Extractions were sent on dry ice to BGI (San Jose, CA) for shotgun sequencing. We were unable to sequence two samples due to low DNA concentration (RRMS, *n* = 48). An aliquot of each original saliva sample was also shipped on dry ice to Metabolon (Morrisville, NC) for metabolite identification.

### Quality control

For DNA extraction, we included a water control to monitor for potential contamination. Sequencing was conducted by the commercial provider BGI (Now Innomics, Sunnyvale, California), which implements rigorous quality control measures. Besides checking for sample integrity using agarose gel electrophoresis, BGI used SOAPnuke^52^, a MapReduce-accelerated tool for quality control and preprocessing of high-throughput sequencing data.

During preprocessing, reads were removed if they met any of the following criteria: (1) ≥ 25% adapter content (allowing up to three mismatches), (2) length <150 bp, (3) ≥ 0.1% ambiguous ‘N’ bases, or (4) ≥ 50% of bases with a quality score <20. The remaining high-quality reads were retained using a Phred+64 quality system. In our samples, >91% of reads had a Q30 or higher score, and over 97% had Q20 or higher score, ensuring high data reliability for downstream analyses.

### Metagenomic profiling

Raw sequence files were processed using the *metaWRAP*^53^ pipeline (assess read quality, trim adapters, and remove human sequences). We used *KRAKEN2*^54^ to identify taxonomy followed by *BRAKEN*^55^ for abundance refinement. Lastly, we used *bit*^56^ to combine taxonomy abundances and add lineage.

We also used the *metaWRAP* pipeline to identify the functional capabilities of the oral bacteriome. For assembly we utilized *metaSPAdes*^57^, binning was performed with *MetaBAT2*^58^*, MaxBin2*^59^, and *CONCOCT*^60^, bin refinement and reassembly was performed with the provided *metaWRAP* algorithms and bin quantification was performed with *salmon*^61^. Final functional annotation was performed with *prokka*^62^ and pathway construction with *MinPath*^63^.

### Salivary metabolomic profiling

Untargeted metabolomics was performed by Metabolon Inc. (Morrisville, NC) using proprietary methods as previously described by our group^64,65^. In brief, Metabolon Inc. utilizes reverse-phase ultra-high-performance chromatography-mass spectrometry with negative and positive ion mode electrospray ionization (ESI) and hydrophilic interaction ultra-performance liquid chromatography-mass spectrometry with negative ion mode ESI. Metabolon ran several controls along with our samples to assure quality measurements, including a pooled sample containing a small component of each of our samples, ultra-pure water, a mixture of solvents used in the extraction, and a well-characterized sample maintained by Metabolon Inc. Compounds were then identified based on their retention time/index (RI), mass to charge ratio (m/z), and fragmentation characteristics (MS/MS spectral data) by comparison to Metabolon’s comprehensive library of purified standard compounds. A total of 964 metabolites were identified (Supplementary Data 2 and 3).

### Microbiome data analysis

There were 2933 raw ASVs at the start of analysis. On average RRMS samples had 699,160 reads (min: 226,572; max: 935,052) and HCs had 693,575 reads (min: 283,382; max: 845,028). All of the following data analysis was performed in R (version 4.3.1). *Phyloseq* (version 1.44.0)^66^ was utilized for filtering, normalization, and data formatting. We filtered our bacterial abundance table to maintain features with at least an abundance of 30 across 50% of samples, resulting in 249 species for analysis. For the viral component, RRMS and HC samples had 1253 and 1634 reads on average, respectively. There were 194 viruses and post filtering ( ≥ 10 in 50% of samples), 15 remained. As for the fungi, RRMS and HC samples had 1495 and 1503 reads on average, respectively. There were 84 fungi and post filtering ( ≥ 10 in 50% of samples), 49 remained.

The filtered abundance tables were then normalized by applying the centered log-ratio (CLR) or total sum scaling (TSS) to 1e6 followed by a log10 transformation. Raw data was used for alpha diversity analyses (Chao1, Shannon, and PD), TSS/log data for beta diversity (Weighted UniFrac, ADONIS from the vegan package (version 2.6-4)^67^ for significance), and CLR data for beta diversity (Aitchison, ANOSIM from the vegan package (version 2.6-4)^67^ for significance) and univariate analyses (Wilcoxon Rank Sum Test). We applied the Benjamini Hochberg (BH) Procedure to account for multiple testing when using the Wilcoxon Test and an FDR (q-value) of 0.05 was considered significant. All figures were created with *ggplot2* (version 3.4.2)^68^.

For bacterial community assessment we utilized Topic Modeling, an unsupervised machine learning approach to identify themes and patterns within data as described previously by our group^29^. Briefly, Topic Modeling offer the advantages of grouping bacteria into meaningful community patterns, with each ‘topic’ representing a potential bacterial community. First we choose optimal number of topics using metrics developed by CaoJuna2009^69^ and Arun2010^70^. We took our filtered phyloseq object and identified the ideal topic number using *FindTopicsNumber* from the *ldatuning* package (version 1.0.2)^71^. *X*-axis on the graph represent number of topics whereas y-axis represents the minimization score calculated by each metric based on the topic number (Fig. 3A). Using the *LDA* function from the *topicmodels* package (version 0.2.14)^31,72^ we performed Latent Dirichlet Allocation on our abundance table with *k* = *33* and *method* = *“VEM”*. Then with the *tidyverse* package (version 2.0.0)^73^, we extracted the beta and gamma matrices for analysis of sample and species community assignment.

183 functional pathways were identified and post-filtering ( ≥ 10 in 50% of samples) 48 remained for analysis. Pathways were normalized with TSS to 1e6. The Wilcoxon Test and BH procedure were utilized to identify individual metabolite changes and the same significance cut-offs were applied. Radar plots were created with *ggradar* (version 0.2)^74^ and functional names were translated with MetaCyc^75^.

### Cohort covariates

Our RRMS cohort had a significantly higher BMI and female/male ratio compared to HCs. We performed linear regression to assess if these covariates made a significant impact on the oral microbiome or metabolome of our cohort. Specifically, our first regression had two explanatory variables, group (RRMS/HC) and BMI (*lm(bacteria_abundance ~ group* + *BMI)*). After adjusting for multiple comparisons, we found that no species abundances were significantly altered by BMI (*q* ≤ 0.05). We found the same results when also providing an interaction term *lm(bacteria_abundance ~ group* + *BMI* *+* group*BMI). The same analysis was performed for the oral metabolome, and no metabolites were significantly altered by BMI.

Although RRMS is more often diagnosed in women than men we wanted to assess the impact of sex on the oral microbiome and metabolome as our female to male ratio was higher in the RRMS cohort compared to HCs. We repeated our linear regression analysis with *sex* as an explanatory variable and found the same results. No species were significantly altered by sex. Additionally, one HC was “intersex” and because we are not able to perform this analysis with *n* = 1, we removed this sample from further analysis (HC, *n* = 49). This analysis was also performed on the oral metabolome and one metabolite was significantly altered based on sex, 2-amino-4-cyanobutanoate, however this metabolite was not significantly different between pwRRMS and HCs in our univariate analysis.

We performed alpha and beta diversity analyses on our RRMS cohort to assess smoking and drug history impact on the oral microbiome. Comparing the RRMS non-smokers with the RRMS patients that quit smoking 10 or more years ago we found that the alpha diversity (Supplementary Fig. 3A), did not significantly vary. Additionally, beta diversity (Weighted UniFrac and Aitchison) was not significantly different between the smoking groups (Supplementary Fig. 3B). As for variation in drug treatment, alpha and beta diversity were not significantly different between patients on different drug therapies (Supplementary Fig. 3C, D). To assess the metabolome, we used PCA and did not find significant differences between RRMS patients based on smoking history or drug therapy (Supplementary Fig. 3E, F).

### Metabolome data analysis

One sample was removed due to low abundance of saliva. This sample was from a female in the RRMS cohort, slightly lowering the BMI and age means for the metabolome analysis (RRMS = 47, BMI = 28.61 ± 5.19, Age = 41.87 ± 8.67). Of the 964 identified metabolites, 775 were known. The metabolites were batch normalized by mean, imputed (for each metabolite, a missing value was replaced with its observed minimum), and log transformed. All the following data analyses were performed in R and figures were created with *ggplot2*. OPLS-DA model was built with HC = 25 and RRMS = 24 and tested on the remaining data. PCA plot utilized Euclidean distance and ANOSIM for significance calculation. To identify significant metabolites the Wilcoxon and BH procedure were again applied with the same significance cut-offs as the microbiome analysis.

To predict the origin of the metabolites, we utilized AMON (Annotation of Metabolite Origin via Networks, version 1.0.4)^37^. Roughly 60% of our identified species had a corresponding KEGG organism ID and approximately 40% of metabolites had a compound ID (labeled by Metabolon). We downloaded the homo sapiens KO’s directly from KEGG. To identify the modules impacted by the microbiome, we provided the KEGG compound ID’s of our microbial metabolites to pathways.embl.de^76^.

### Random Forest analysis of metabolome

We utilized the randomForest (version 4.7.1.1)^77^ and Boruta (version 8.0.0)^78^ packages to perform Random Forest, a supervised machine learning classification technique. Our training data was roughly 75% of our cohort (RRMS = 38, HC = 37). We built our Random Forest model with 4000 trees. Our model was tested on the remaining cohort (RRMS = 9, HC = 12). To confirm important features, we then applied Boruta, where the important features are compared to shadow features (randomly permutated copies of the original features), with the suggested significance level of 0.01 and for 100 iterations.

### Correlation of the oral microbiome and metabolome

Spearman’s correlation (which captures monotone associations, in contrast to linear associations from Pearson’s correlation) was computed for each combination of the selected normalized and filtered bacteria (described under Microbiome data analysis) and the normalized and filtered metabolites (described in in Metabolome data analysis). The “*q*” value refers to Benjamini Hochberg Adjusted *p* values, so the “*p*” value threshold can be omitted so that the sentence just reads: “A Spearman’s correlation FDR-adjusted *p* value of <0.05 was considered significant”.

## Supplementary information

Supplementary information

Supplementary Data 1

Supplementary Data 2

Supplementary Data 3

## Data Availability

All raw shotgun metagenomic sequences can be found at the Sequence Read Archive (SRA) under the BioProject ID PRJNA1090491 for free public access.

## Code availability

All code and corresponding metadata can be found at the public GitHub repository: https://github.com/raeshrode/Oral_Microbiome_MS.
