# Supplementary material for: Multiple sclerosis patients exhibit oral dysbiosis with decreased early colonizers and lower hypotaurine level
Source: NPJ Biofilms Microbiomes. 2025 Oct 20;11:199. doi: 10.1038/s41522-025-00787-7 (PMC12537959; doi:10.1038/s41522-025-00787-7)
Supplement: Supplementary file 1 — Supplementary information [file 41522_2025_787_MOESM1_ESM.pdf]

Supplementary Figures and Tables

Supplementary Figure 1: The virome and mycobiome vary in the oral microbiome of RRMS patients

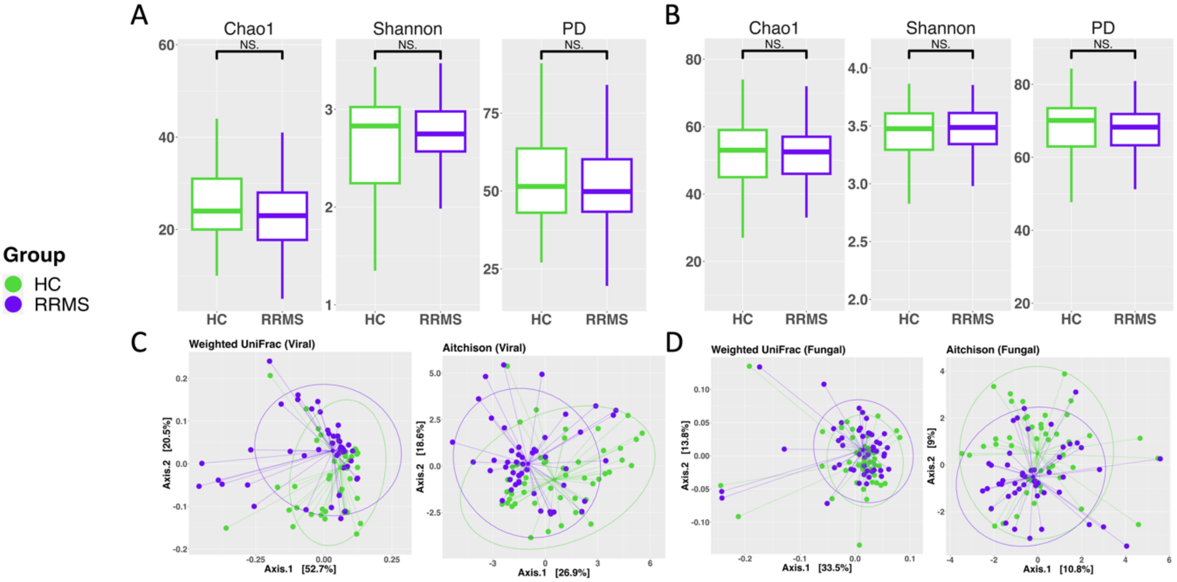

Supplementary Figure 1. Alpha diversity comparison of the oral microbiome in pwRRMS compared to HCs when considering the **A**, viral component and the **B**, fungal component. Beta diversity comparison, using Weighted UniFrac (left) and Aitchison (right), between pwRRMS and HCs when considering the **C**, viral component and the **D**, fungal component.

Supplementary Figure 2: Hypotaurine and Taurine correlations in healthy controls

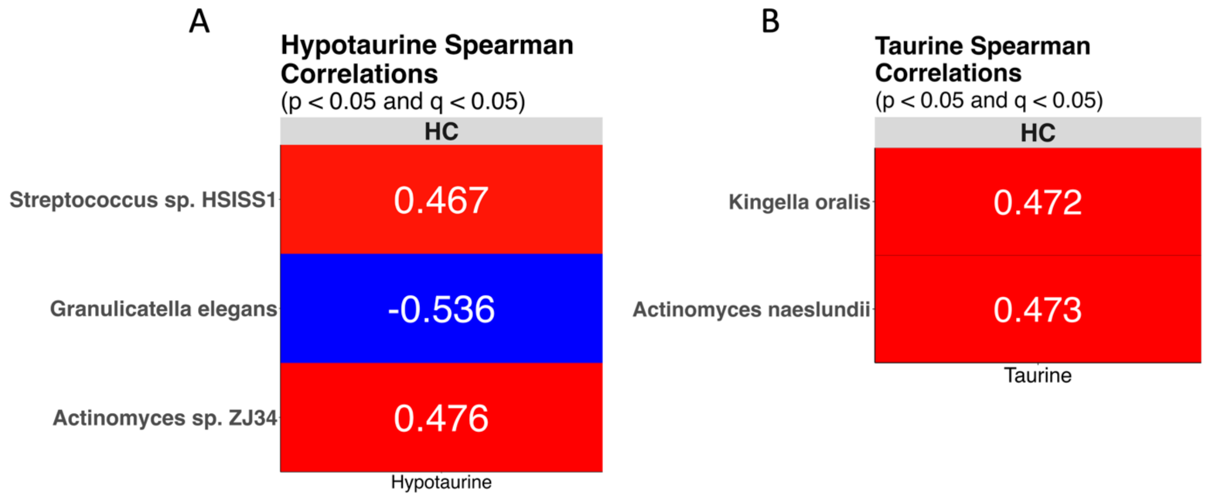

Supplementary Figure 2. **A**, Significant correlations between Hypotaurine and significantly altered bacteria. **B,** Significant correlations between Taurine and significantly altered bacteria. These correlations either did not exist or were not significant in the RRMS cohort.

Supplementary Figure 3: Covariate preliminary analysis of the microbiome and metabolome

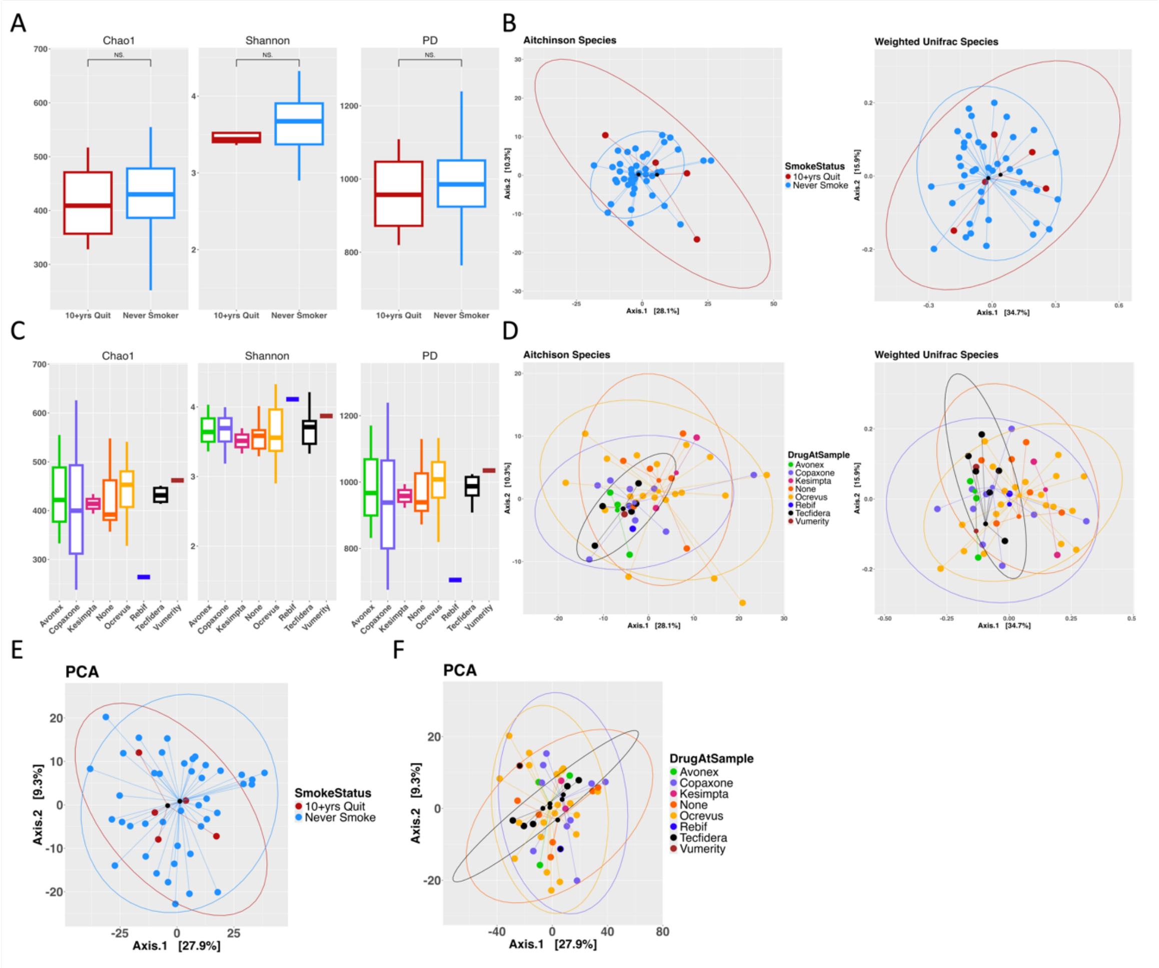

Supplementary Figure 3. Smoking history effect on the oral microbiome of pwRRMS based on **A**, alpha diversity **B**, and beta diversity. Drug therapy effect on the oral microbiome of pwRRMS based on **C**, alpha diversity **D**, and beta diversity. **E,** Smoking history and **F**, drug therapy effects on oral metabolome of pwRRMS.

Supplementary Table 1. Bacterial, viral, and fungal species significantly altered in pwRRMS

Species	p	q	Higher In	Gram
Actinomyces sp. oral taxon 414	8.11E-11	1.88E-08	HC	positive
Pauljensenia hongkongensis	1.01E-08	1.17E-06	HC	positive
Actinomyces howellii	3.47E-08	2.01E-06	HC	positive
Actinomyces radicidentis	2.73E-08	2.01E-06	HC	positive
Actinomyces sp. MAS-1	8.51E-08	3.95E-06	HC	positive
Streptococcus sp. HSISS3	1.29E-07	4.97E-06	HC	positive
Streptococcus salivarius	1.93E-07	5.19E-06	HC	positive
Streptococcus sp. FDAARGOS_192	2.01E-07	5.19E-06	HC	positive
Streptococcus sp. HSISS1	1.84E-07	5.19E-06	HC	positive
Prochlorococcus virus NATL1A7	5.72E-07	8.58E-06	HC	
Olsenella sp. oral taxon 807	1.31E-06	3.05E-05	HC	positive
Fusobacterium hwasookii	2.81E-06	5.93E-05	RRMS	negative
Riemerella anatipestifer	3.16E-06	6.11E-05	RRMS	negative
Actinomyces sp. ZJ750	7.05E-06	0.00012574	HC	positive
Streptococcus sp. HSISS2	1.18E-05	0.0001956	HC	positive
Streptococcus thermophilus	1.27E-05	0.00019629	HC	positive
Streptococcus pseudoporcinus	1.82E-05	0.00026328	HC	positive
Actinomyces sp. ZJ34	2.41E-05	0.00032829	HC	positive
Streptococcus vestibularis	4.76E-05	0.000614	HC	positive
Fusobacterium canifelinum	5.82E-05	0.00071014	RRMS	negative
Streptococcus sp. NSJ-72	7.08E-05	0.00082156	HC	positive
Porphyromonas somerae	7.56E-05	0.0008351	RRMS	negative
Actinomyces sp. oral taxon 897	8.07E-05	0.00085055	HC	positive
Streptococcus sp. I-G2	9.47E-05	0.00095566	HC	positive
Capnocytophaga endodontalis	0.0001111	0.00107397	RRMS	negative
Actinomyces sp. HMT 897	0.00012214	0.0010899	HC	positive
Streptococcus milleri	0.00012214	0.0010899	HC	positive
Bacteroides uniformis	0.0001342	0.00115316	RRMS	negative
Escherichia virus N4	0.0002332	0.00131454	HC	
Pseudomonas phage PPpW-3	0.00026291	0.00131454	HC	
Streptococcus koreensis	0.00017201	0.00142522	HC	positive
Leptotrichia sp. oral taxon 212	0.00018858	0.00150866	RRMS	negative
Actinomyces naeslundii	0.00020663	0.0015464	HC	positive
Streptococcus constellatus	0.00020663	0.0015464	HC	positive
Streptococcus parasanguinis	0.0002332	0.00169068	HC	positive
Streptococcus lactarius	0.00033314	0.00234204	HC	positive

Species	p	q	Higher In	Gram
Corynebacterium matruchotii	0.00040844	0.00278703	HC	positive
Porphyromonas sp. oral taxon 275	0.00045826	0.00303758	RRMS	negative
Fusobacterium periodonticum	0.00047156	0.00303892	RRMS	negative
Eikenella corrodens	0.0005284	0.00322602	RRMS	negative
Parabacteroides distasonis	0.0005284	0.00322602	RRMS	negative
Bacteroides zoogleoformans	0.00057511	0.00342117	RRMS	negative
Cutibacterium acnes	0.0007189	0.0040679	HC	positive
Granulicatella elegans	0.0007189	0.0040679	RRMS	positive
Streptococcus sanguinis	0.0008951	0.00494436	HC	positive
Actinomyces sp. oral taxon 169	0.00111016	0.00562788	HC	positive
Actinomyces sp. zg-993	0.00114013	0.00562788	HC	positive
Schaalia meyeri	0.00114013	0.00562788	HC	positive
Schaalia odontolytica	0.00114013	0.00562788	HC	positive
Streptococcus sp. HSISM1	0.0010809	0.00562788	HC	positive
Streptococcus cristatus	0.00152235	0.00735802	HC	positive
Aeromonas virus 65	0.00196765	0.00737869	RRMS	
Actinomyces viscosus	0.00173202	0.00820057	HC	positive
Fusobacterium pseudoperiodonticum	0.00191832	0.00890099	RRMS	negative
Streptococcus sp. LPB0220	0.00201814	0.00918055	HC	positive
Prevotella sp. oral taxon 299	0.00228867	0.01021097	RRMS	negative
Streptococcus anginosus	0.00234656	0.01027176	HC	positive
Prevotella sp. oral taxon 475	0.00279047	0.01198869	RRMS	negative
Haemophilus sp. oral taxon 036	0.00285974	0.01206289	RRMS	negative
Prevotella copri	0.00293055	0.01214086	RRMS	negative
Prevotella ruminicola	0.00315261	0.01283168	RRMS	negative
Alloprevotella sp. E39	0.00338974	0.01355897	RRMS	negative
Streptococcus pyogenes	0.00355664	0.01398543	HC	positive
Bacteroides xylanisolvens	0.00364283	0.01408562	RRMS	negative
Veillonella rogosae	0.00382088	0.01453188	RRMS	positive
Capnocytophaga sp. oral taxon 878	0.00391281	0.01464148	RRMS	negative
Streptococcus phage EJ-1	0.0050634	0.01519019	RRMS	
Streptococcus rubneri	0.00461397	0.01699114	HC	positive
Streptococcus equi	0.00472284	0.0171203	HC	positive
Haemophilus influenzae	0.0050634	0.01807243	RRMS	negative
Fusobacterium nucleatum	0.00518171	0.01821448	RRMS	negative
Capnocytophaga sputigena	0.00530248	0.01836083	RRMS	negative
Haemophilus haemolyticus	0.00581114	0.01982626	RRMS	negative
Prevotella sp. Rep29	0.00608145	0.02044779	RRMS	negative

**Species**	**p**	**q**	**Higher In**	**Gram**
Schaalia cardiffensis	0.00760836	0.02385325	HC	positive
Streptococcus mutans	0.00760836	0.02385325	HC	positive
Neisseria sp. ZJ785	0.00760836	0.02385325	RRMS	negative
Porphyromonas gingivalis	0.00727824	0.02385325	RRMS	negative
Prevotella intermedia	0.00744168	0.02385325	RRMS	negative
Streptococcus australis	0.00777835	0.02406104	HC	positive
Neisseria weaveri	0.0090656	0.02767393	RRMS	negative
Prevotella corporis	0.00926409	0.02791258	RRMS	negative
Capnocytophaga sp. FDAARGOS_737	0.01053767	0.03094606	RRMS	negative
Eikenella exigua	0.01053767	0.03094606	RRMS	negative
Sugiyamaella lignohabitans	0.00064331	0.03152241	RRMS	
Neisseria sp. 10022	0.01099521	0.0318861	RRMS	negative
Schaalia turicensis	0.01147014	0.03285273	HC	positive
Kingella oralis	0.01171429	0.03314287	RRMS	negative
Bacteroides fragilis	0.01247436	0.03486808	RRMS	negative
Streptococcus sp. oral taxon 431	0.01383745	0.03810515	HC	positive
Capnocytophaga leadbetteri	0.01412518	0.03810515	RRMS	negative
Prevotella buccalis	0.01412518	0.03810515	RRMS	negative
Capnocytophaga sp. oral taxon 902	0.01441813	0.03844834	RRMS	negative
Capnocytophaga ochracea	0.01501997	0.03959812	RRMS	negative
Neisseria flavescens	0.0162897	0.04246304	RRMS	negative
Neisseria zalophi	0.01662136	0.04284617	RRMS	negative
Lachnoanaerobaculum umeaense	0.01730233	0.04411144	HC	positive
Bacteroides heparinolyticus	0.01800741	0.04540998	RRMS	negative
Prevotella dentalis	0.01836919	0.04582423	RRMS	negative
Streptococcus intermedius	0.01911171	0.04716933	HC	positive
Actinomyces sp. HMT 175	0.02067499	0.04996456	HC	positive
Streptococcus agalactiae	0.02067499	0.04996456	HC	positive

Supplementary Table 2. AMON results			
**Microbiome**		**Human**	
CHEMICAL_NAME	KEGG	CHEMICAL_NAME	KEGG
tryptophan	C00078	N-acetylneuraminate	C00270
phenylalanine	C00079	carnosine	C00386
ribose	C00121	spermine	C00750
malate	C00149	cortisone	C00762
lactate	C00186	N-acetylaspartate (NAA)	C01042
threonine	C00188	3-methylhistidine	C01152
oxalate (ethanedioate)	C00209	1-methylhistidine	C01152
butyrate/isobutyrate (4:0)	C00246	3-hydroxyisobutyrate	C01188
prephenic acid	C00254	fructose 1,6-diphosphate/glucose 1,6-d	C01231
cytosine	C00380	anserine	C01262
malonate	C00383	caprate (10:0)	C01571
mannitol/sorbitol	C00392	hippurate	C01586
5-aminovalerate	C00431	glycocholate	C01921
N-carbamoylputrescine	C00436	creatine phosphate	C02305
glutarate (C5-DC)	C00489	1-methylnicotinamide	C02918
arabonate/xylonate	C00502	dihomo-linolenate (20:3n3 or n6)	C03242
4-cholesten-3-one	C00599	androstenediol (3beta,17beta) disulfate	C04295
gentisate	C00628	dehydroepiandrosterone sulfate (DHEA	C04555
diaminopimelate	C00680	1-methylhistamine	C05127
lysine	C00739	N6,N6-dimethyllysine	C05545
salicylate	C00805	N1-methyl-2-pyridone-5-carboxamide	C05842
galactonate	C00880	caprylate (8:0)	C06423
1,3-diaminopropane	C00986	arachidate (20:0)	C06425
3-phosphoserine	C01005	docosahexaenoate (DHA; 22:6n3)	C06429
3-hydroxypropanoate	C01013	palmitoleate (16:1n7)	C08362
O-acetylhomoserine	C01077	cyclic adenosine diphosphate-ribose	C13050
trimethylamine N-oxide	C01104	7-methylurate	C16355
galactose 6-phosphate	C01113	1,7-dimethylurate	C16356
2-methylcitrate/homocitrate	C01251	1-methylurate	C16359
adenosine 3'-monophosphate (3'-AMP)	C01267	desmethylcitalopram*	C16608
uridine 3'-monophosphate (3'-UMP)	C01368	cholesterol sulfate	C18043
cadaverine	C01672	beta-citrylglutamate	C20775
3-(4-hydroxyphenyl)propionate	C01744	dimethylarginine (SDMA + ADMA)	C21189
citraconate/glutaconate	C02226	oleate/vaccenate (18:1)	C21944
allantoin	C02348		
alpha-tocopherol	C02477		
2-isopropylmalate	C02504		
3-dehydroshikimate	C02637		

Microbiome		Human	
methionine sulfoxide	C02989		
cytidine 2' or 3'-monophosphate (2' or 3'-CMP)	C03104		
3-hydroxybutyrate (BHBA)	C03197		
deoxythymidine diphosphate-rhamnose	C03319		
N-acetylphenylalanine	C03519		
2,3-dihydroxyisovalerate	C04039		
dehydroascorbate	C05422		
ergothioneine	C05570		
cysteine s-sulfate	C05824		
vanillate	C06672		
theobromine	C07480		
delta-tocopherol	C14151		
gamma-tocopherol/beta-tocopherol	C14152		
N-acetylcitrulline	C15532		
7-methylxanthine	C16353		
3-methylxanthine	C16357		
5-hydroxymethyl-2-furoic acid	C20448		

Supplementary Data 1. Significant correlations between significant bacterial species and metabolites

Supplementary Data 2. Metabolite abundance table

Supplementary Data 3. Metabolite identification table
